# Capillaroscopy and Immunological Profile in Systemic Sclerosis

**DOI:** 10.3390/life12040498

**Published:** 2022-03-29

**Authors:** Sevdalina Nikolova Lambova, Ekaterina Krasimirova Kurteva, Sanie Syuleymanova Dzhambazova, Georgi Hristov Vasilev, Dobroslav Stanimirov Kyurkchiev, Mariela Gencheva Geneva-Popova

**Affiliations:** 1 Department of Propaedeutics of Internal Diseases, Faculty of Medicine, Medical University of Plovdiv, 4002 Plovdiv, Bulgaria; sani_dj@abv.bg (S.S.D.); genevapopova@yahoo.com (M.G.G.-P.); 2 Department of Rheumatology, MHAT “Sveti Mina”, 4000 Plovdiv, Bulgaria; 3 Laboratory of Clinical Immunology, University Hospital “St. Ivan Rilski”, 1431 Sofia, Bulgaria; katrinkrasimirova@gmail.com (E.K.K.); georgihristovvasilev1991@gmail.com (G.H.V.); dsk666@gmail.com (D.S.K.); 4 Department of Clinical Immunology, Faculty of Medicine, Medical University of Sofia, 1431 Sofia, Bulgaria; 5 Clinic in Rheumatology, UMHAT “Sveti Georgi”, 4000 Plovdiv, Bulgaria

**Keywords:** capillaroscopy, systemic sclerosis, autoantibodies

## Abstract

Introduction: Data on the associations between capillaroscopic changes and diagnostic systemic-sclerosis (SSc)-related antibodies are scarce. Presence of such correlation would improve current knowledge about the disease’s pathogenesis by revealing the mechanisms of microangiopathy. The microvascular pathology of SSc is a hallmark of the disease, and immunological abnormalities probably contribute to its development. Patients and methods: 19 patients with definite diagnosis of SSc were included in the current pilot study; 16 had limited and 3 had diffuse cutaneous involvement; their mean age was 51.56 ± 15.07 years. All patients exhibited symptoms of Raynaud’s phenomenon of the fingers. A “scleroderma” type capillaroscopic pattern was classified according to the staging suggested by Cutolo et al. (2000): “early”, “active” or ”late” phase. In the presence of different degrees of capillaroscopic changes in different fingers, the most-advanced microvascular pathology was chosen for classification. In cases without capillaroscopic features of microangiopathy, the findings were categorized as normal or nonspecific (dilated, tortuous capillaries, and/or hemorrhages). Indirect immunofluorescence on HEp-2 cells was performed as the gold-standard screening method for the detection of antinuclear autoantibodies (ANA), and determination of the immunofluorescent staining pattern (anti-cell pattern) was in accordance with the International Consensus on ANA Patterns. Scleroderma-associated autoantibodies in the patients’ serum were assessed using line immunoblot assay for detection of autoantibodies to 13 scleroderma-associated autoantigens: Scl-70, CENP A, CENP B, RP11/RNAP-III, RP155/RNAP-III, fibrillarin, NOR-90, Th/To, PM-Scl100, PM-Scl75, Ku, PDGFR, and Ro-52. Results: In 73.7% (*n* = 14) of the examined patients, “scleroderma” type capillaroscopic changes were found, and in 26.3% (*n* = 5), capillaroscopic features of microangiopathy were absent (nonspecific changes, *n* = 3; normal findings, *n* = 2). In SSc patients with positive anti-Scl-70 (*n* = 7) antibodies, significantly lower mean capillary density was observed along with a higher frequency of “active” and “late” phase capillaroscopic changes as compared to the anti-Scl-70-negative patients (*p* < 0.05). Anti-RNAP III–155 positive patients (*n* = 4) had significantly higher mean capillary density than anti-RNAP III–155 negative patients (*n* = 15). In three of the anti-RNAP III–155-positive cases, capillaroscopic features of microangiopathy were not detected, and in one case there was an “early” phase “scleroderma” pattern. Conclusion: In the current pilot study, the association between more advanced capillaroscopic changes and the presence of anti-Scl-70 autoantibodies was confirmed. As a novel observation, positive anti-RNAP III–155 antibodies were found in SSc patients with or without early microangiopathy. The question of associations between microvascular changes in SSc and other SSc-related autoantibodies requires further research.

## 1. Introduction

Systemic sclerosis (SSc) is a connective tissue disease of unknown etiology and unique immunopathogenesis. It involves presence of microangiopathy, abnormalities of the innate and adaptive immune system (leading to the production of a wide array of autoantibodies and cell-mediated autoimmunity), and fibroblast activation (leading to fibrosis of the skin and internal organs) [1]. Scleroderma-associated antibodies and capillary alterations in the nailfold area have been established as diagnostic criteria. Three autoantibodies are included in the current EULAR (European League Against Rheumatism)/ACR (American College in Rheumatology) classification criteria for SSc: anti-centromere, anti-Scl-70, and anti-RNA-polymerase III (anti-RNAP III) [2,3].

Microvascular changes in the nailfold area in SSc are termed “scleroderma” type capillaroscopic pattern and are characterized by the presence of giant capillaries, hemorrhages, avascular areas/devascularization, and ramified capillaries/neoangiogenesis. They are observed in 70% to more than 90% of patients with overt SSc [4,5,6,7,8,9,10]. In the 80s, Maricq et al. suggested the existence of two major categories of capillaroscopic changes in SSc: “active” and “slow” capillaroscopic patterns. These terms reflect an association with disease activity and progression. The “active” capillaroscopic pattern is characterized by advanced devascularization and neoangiogenesis and has been thought to be an indicator of high disease activity and worse prognosis. The “slow” pattern is characterized by a high number of megacapillaries and mild capillary loss and is an indicator of decreased disease activity [5]. Presence of isolated capillary dilations were marked as an “early” capillaroscopic pattern [9].

The classification of the “scleroderma” type capillaroscopic changes of Cutolo et al. has been validated and is presently widely used. According to this staging system, there are three distinct phases: “early”, “active”, and “late.” The “early” phase is characterized by the appearance of a few giant capillaries; a few microhemorrhages may also be present, and capillary density and architecture are unchanged. In the “active” phase, there are frequent giant capillary loops and microhemorrhages associated with moderate devascularization and capillary derangement. In the “late” phase, capillary loss is advanced, and there is also severe capillary derangement and newly formed, neoangiogenic capillaries with atypical morphology [10]. Here, the terminology reflects the concept of the consecutive appearance of capillaroscopic changes in evolution of the disease.

Association between capillaroscopic changes and immunological alterations in SSc is interesting and insufficiently studied. A correlation between the presence of anti-Scl-70 antibodies and more advanced microvascular changes (i.e., “active” and” late” phases) has been observed [11]. In 241 SSc patients, Cutolo et al. (2004) detected anti-Scl-70 antibodies in 5%, 25%, and 24% of cases with the “early”, “active”, and “late” patterns, respectively. Anti-Scl-70 antibody in the “early” pattern was less frequent compared to both “active” and “late” patterns (*p* < 0.01). A correlation between positive anti-Scl-70 antibody and the duration of Raynaud’s phenomenon (RP) and SSc has not been observed. Regarding anti-centromere antibodies, no statistical difference in frequency was observed between the three capillaroscopic patterns (“early”, “active”, and “late”). Of note, significant correlation was found between anti-centromere antibody positivity and the duration of both RP and SSc (*p* < 0.03). The significantly higher prevalence of anti-Scl70 antibodies in “active” and “late” capillaroscopic patterns and the absence of correlation between these antibodies and the duration of either RP or SSc has led to the conclusion that the presence of anti-Scl-70 antibodies is probably an antecedent event that might be related to the earlier appearance of more-advanced microvascular changes (i.e., “active” and “late” patterns). Due to the higher prevalence of anti-centromere antibodies in patients with longer RP duration, it has been suggested that their presence might be related to delayed expression of the “late” pattern [11].

In a large patient population from the EUSTAR database (1870 patients) with capillaroscopic staging data available, correlation has also been observed between anti-Scl-70 antibodies and “late” phase capillaroscopic changes [12].

Similar are the observations of van Leeuwen et al. (2021) in 164 SSc patients—they found anti-topoisomerase antibodies more frequently in cases with more severe micro-angiopathy, as assessed via nailfold videocapillaroscopy. In SSc with positive anti-centromere antibodies of the IgG class, less-severe microangiopathy was present as compared to patients also expressing IgM and IgA anti-centromere antibodies [13]. Similarly, Pizzorni et al. found significant associations between the “late” capillaroscopic pattern and the presence of anti-Scl-70 antibodies in 33 SSc patients. The advanced “late” type miscovascular changes also correlated to disease duration of at least 5 years and the presence of digital ulcers. Anti-centromere antibodies were associated with milder capillaroscopic changes (i.e., “early” and “active” type). Of note, no statistically significant associations were found between total antinuclear antibodies (ANA) (indirect immunofluorescence (IIF) of HEp-2 cells). However, among patients with severe microangiopathy, all tested positive for ANA [14]. Chen et al. (1984) observed anti-centromere antibodies in 44.8% of SSc cases with scleroderma-spectrum disorders and “slow” capillaroscopic pattern (according to the definition of Maricq et al.) and in 9.7% of those with a normal pattern. In cases with an “active” pattern (according to the Maricq definition), anti-centromere antibodies were not detected [15]. However, in an Italian patient population of 103 SSc patients, Caramaschi et al. (2007) did not find an association between positivity for ANA, anti-centromere antibodies, anti-Scl-70 antibodies and capillaroscopic “early”, “active”, and “late” patterns [16]. In a recent systematic review, van Leeuwen et al. (2021) concluded that the data available regarding the association between auto-antibodies and microangiopathy in SSc are not unambiguous [17].

Improvement of immunological diagnostics has shown that for patients in whom the diagnostic SSc-specific autoantibodies are negative, other SSc-associated autoantibodies could be found, which, while not included in the current classification criteria, have diagnostic potential. The extended profile of SSc-associated autoantibodies is already broadly used in clinical practice. EUROLINE Systemic Sclerosis immunoblot has been approved for screening of 13 scleroderma-specific or associated antibodies: anti-Scl-70, CENPA, CENPB, RNAP-III (11 kDa), RNAP-III (155 kDa), fibrillarin (U3-RNP), NOR90, Th/To, PM/Scl-100. PM/Scl-75, Ku, PDGFR, and Ro52 [18]. Data on their associations with capillaroscopic changes in SSc are scarce [19,20].

In conclusion, analysis of the available literature data shows that associations between capillaroscopic changes and immunological profile in SSc require further research. Most of the studies on this topic are about the associations of anti-Scl-70 and anti-centromere antibodies. Associations between capillaroscopic changes and other SSc-associated antibodies or total ANA are insufficient.

## 2. Materials and Methods

A total of 19 patients with definite-diagnosis SSc were included in the current pilot study: 1 male and 18 females; 16 with limited and 3 with diffuse cutaneous involvement; mean age 51.56 ± 15.07 years. RP of the fingers was present in all patients.

After acclimatizing the patients for 15–20 min at room temperature (around 25 °C), capillaroscopic examination was performed at 8 fingers (II ÷ V bilaterally) using a Mediscope videocapillaroscope (Optilia, Sweden) with magnification at 200x. A drop of paraffin oil was applied in the nailfold area, and four images were obtained for each examined finger.

The presence of standard capillaroscopic parameters was analyzed, i.e., giant capillaries, hemorrhages, neoangiogenesis, and mean capillary density. Quantitative analysis of capillaroscopic images was performed. The accepted capillaroscopic definitions of German-speaking authors (Schmidt et al., 1997) have been used. Capillaries with a diameter of the arterial limb greater than 15 µm (0.015 mm) or venous limb greater than 20 µm (0.020 mm) were classified as dilated. In the presence of capillary diameter greater than 50 µm (0.050 mm), microvessels were defined as giant capillary loops/megacapillaries. The mean capillary density was calculated as the number of capillary loops in the distal row per 1 mm. The normal range is from 7 to 16 capillaries per mm. The avascular areas were defined as the distance between 2 adjacent capillary loops from the distal rows greater than 500 µm (0.500 mm) or greater than 300 µm (0.300 mm) in the proximal area. Presence of more than 1–3 capillary loops that originate from the same vessel in a single dermal papilla, ramified and bushy capillaries are the characteristic features of neoangiogenesis [21].

In cases with “scleroderma” type capillaroscopic pattern, the changes were classified according to the staging suggested by Cutolo et al. (2000): “early”, “active”, and “late” phase [10].

In cases without capillaroscopic features of microangiopathy, the findings were determined as normal or nonspecific findings (dilated, tortuous capillaries, or hemorrhages). Considering the high prevalence of inhomogeneity in SSc, in the presence of different degrees of capillaroscopic changes between fingers, the most-advanced microvascular pathology was chosen [22].

The following immunological techniques were used to determine the presence of scleroderma-associated autoantibodies:

1. We performed IIF on HEp-2 cells (IIFT: HEp-2, Biosystems, Spain) as the gold-standard screening method for the detection of ANA and determination of the immunofluorescent staining pattern (anti-cell pattern (AC)) according to the International Consensus on ANA Patterns (ICAP, https://www.anapatterns.org/31.01.2022, accessed on 31 January 2022). ANA titer 1:160 or higher was considered positive.
2. A mixed pattern consisted of a 5-element (subcellular regions) compound staining pattern AC-29, Topo I-like pattern:
  (1) Prominent, fine-speckled AC-4 type nuclear staining in interphase cells.
  (2) Consistent, strong, fine-speckled staining of the condensed chromatin in mitotic cells that appeared homogeneous at the initial dilution 1:80.
  (3) Strong staining of nucleolar organizing region (NOR) associated with condensed chromosomes in mitotic cells. The NOR staining at dilution 1:80 was obscured by the bright homogeneous chromosomal staining.
  (4) Weak cytoplasmic staining in interphase (and mitotic) cells, depicting a delicate network radiating from the perinuclear area towards the plasma membrane.
  (5) A nucleolar staining that appeared as a punctate nucleolar staining in interphase cells.
3. We analyzed the patients’ serum for the presence of SSc-associated autoantibodies using a commercial line immunoblot assay (Scleroderma (Nucleoli) Profile Euroline (IgG); Euroimmun). The line blot detects autoantibodies to 13 scleroderma-associated autoantigens: Scl-70, CENP A, CENP B, RP11/RNAP-III, RP155/RNAP-III, fibrillarin, NOR-90, Th/To, PM-Scl100, PM-Scl75, Ku, PDGFR, and Ro-52. A single operator performed the assays and analyses per the manufacturer’s instructions. We considered the positive and negative results as defined by the assay, and we reported results in the borderline range as negative.

### Statistical Processing and Study Design

Statistical analyses of data were performed using RStudio (version 1.4.1106–5). The variables describing the capillaroscopic parameters, the ANA titer and ANA staining pattern, and the presence of SSc-specific autoantibodies were categorical. Along this line of thought, Fisher’s exact test was the most appropriate method. Fisher’s exact test is a non-parametric method for comparing the proportion of categories in two different independent groups (categorical or nominal variables) in a contingency table. Fisher’s exact test is an exact test (returns exact *p*-value) and can be applied on smaller sample sizes, especially when the frequency count is <5 for more than 20% of cells.

## 3. Results

Patient characteristics are presented in Table 1.

**Figure 1:**
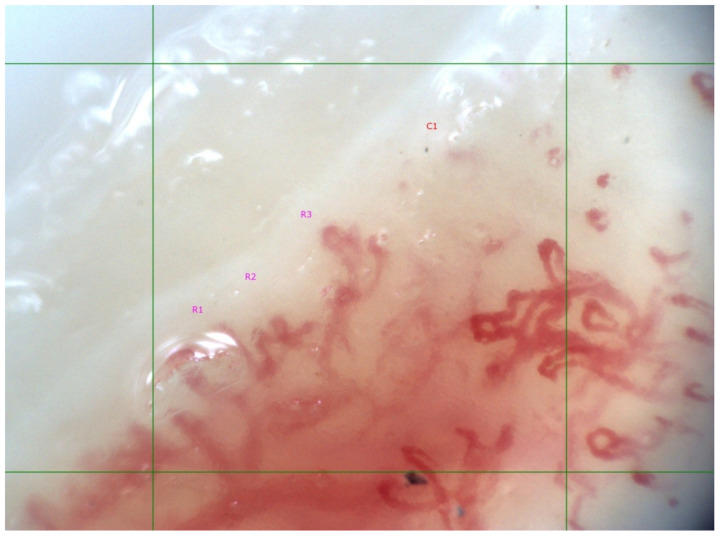
“Scleroderma” type capillaroscopic pattern, “late” phase in 60-year-old female SSc patient with limited cutaneous involvement; anti-Scl-70 antibody positive. Disease duration of 5 years. Mean capillary density is 4/mm (R1, R2, R3—three ramified capillary loops, C1—capillary loop). Ramified/neoangiogenic capillaries and capillary derangement is present.

In 73.7% (*n* = 14) of the examined patients, “scleroderma” type capillaroscopic changes were found, and in 26.3% (*n* = 5), capillaroscopic features of microangiopathy were absent (nonspecific changes, *n* = 3; normal findings, *n* = 2). In SSc patients with positive anti-Scl-70 (*n* = 7) autoantibodies, significantly lower mean capillary density was observed, as well as a higher frequency of “active” and “late” phase capillaroscopic changes as compared to the patients negative for anti-Scl-70 antibodies (*p* < 0.05) (Figure 1). All of the patients with positive anti-Scl-70 autoantibodies had high ANA titers (set at >1:1280), and, according to ICAP recommendations, the staining pattern (AC pattern) was defined as a mixed pattern consisting of a 5-element (subcellular regions) compound staining pattern AC-29, Topo I-like pattern.

**Figure 2:**
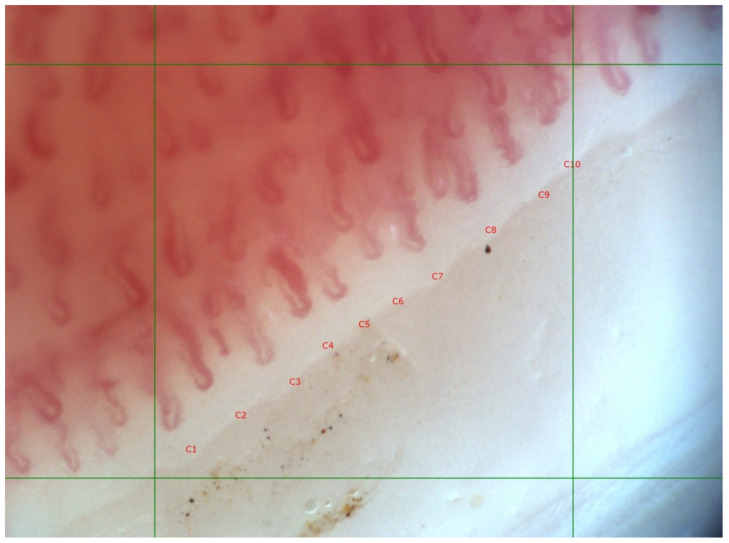
“Scleroderma” type, capillaroscopic pattern, “early” phase in 57-year-old female SSc patient with limited cutaneous involvement, anti-RNAP III–155 antibody positive. Disease duration of 13 years. Mean capillary density 10/mm (C1–C10—ten capillary loops), preserved distribution, presence of dilated and single giant capillaries (C4); magnification 200×.

**Figure 3:**
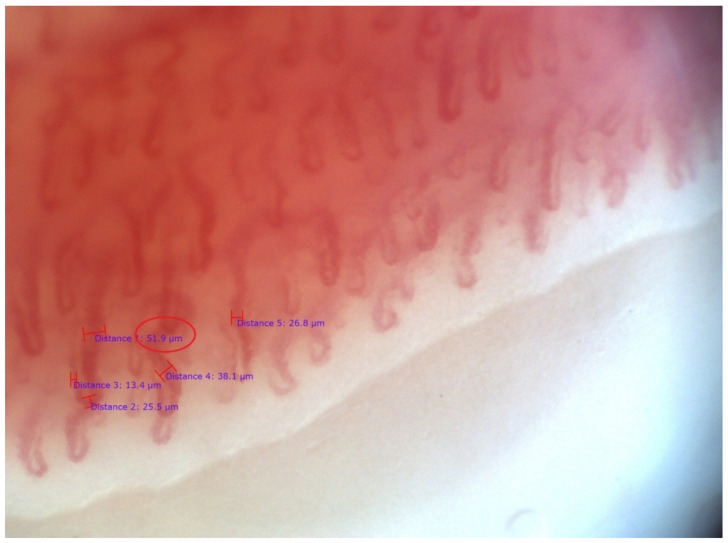
“Scleroderma” type, capillaroscopic pattern, “early” phase in the same patient presented at Figure 2. Measurement of capillaroscopic diameters is demonstrated. There are dilated and a single giant capillary loop with venous diameter 51.9 µm (0.051 mm; circle); magnification 200×.

SSc patients with positive antibodies against RNAP III (epitope155) (*n* = 4) had significantly higher mean capillary density as compared to anti-RNAP III–155 negative cases (*n* = 15). In three of these cases, capillaroscopic features of microangiopathy were not detected, and in one case there was an “early” phase “scleroderma” pattern (Figure 2 and Figure 3). Interestingly, in 3 of the 5 cases without capillaroscopic signs of microangiopathy, anti-RNAP III–155 antibodies were detected, and the ANA titer was set at 1:1280, AC-5, AC-10 pattern. In contrast, in two cases the ANA titer was 1:320, AC-5. Antibodies against both the 155 and 11 RNAP III epitopes were positive in two cases. All other antibodies from the EUROLINE Systemic Sclerosis immunoblot test were negative in all four cases. (Table 2).

There was no statistical correlation between disease duration and ANA titer at the moment of ANA screening. ANA titer 1:1280 was detected in 13 cases. In 5 patients ANA titer was lower (1:640 in a single case, 1:320 in 2 patients, 1:160 in 2 patients), and in one patient ANA were negative (titer 1:80). In the majority of patients with ANA titer 1:1280 (9/13), more-advanced capillaroscopic changes were observed (either “active” (*n* = 6) or” late” pattern (*n* = 3)).

## 4. Discussion

### 4.1. Associations between Capillaroscopic Changes and SSc-Associated Autoantibodies

Improved knowledge about associations between immunological and microvascular abnormalities in SSc is of considerable importance as they are major pathogenic pathways. Moreover, SSc-related autoantibodies are known to be related to certain clinical subsets of the disease [23,24,25,26,27]. In the current pilot study, a previously observed association by other authors between advanced capillaroscopic changes and positive anti-Scl-70 antibodies was confirmed. The presence of anti-Scl-70 antibodies is suggested to contribute to the worsening of microvascular pathology in scleroderma [11,12,14]. Anti-Scl-70 antibodies have shown associations with a higher probability of interstitial lung disease in SSc patients with both diffuse and limited cutaneous involvement and with hand disability and flexion contractures in metacarpophalangeal and proximal interphalangeal joints [25,26,27].

In a group of 287 SSc patients, Markusse et al. assessed the association between the degree of “scleroderma” type microangiopathy (“early”, “active”, or “late”) and extended autoantibody profile of SSc-related autoantibodies (anti-centromere, anti-Scl-70, anti-RNP, anti-RNAP III, anti-fibrillarin, anti-PM/Scl, anti-Th/To, and anti-Ku antibodies). “Active” and “late” capillaroscopic patterns were found with similar frequency in patients positive for anti-Scl-70 antibodies and those positive for anti-centromere, anti-RNP, or anti-RNAPIII antibodies. Thus, it has been concluded that the presence of a specific autoantibody is not associated with the development of microangiopathy. In patients with positive anti-centromere antibodies the risk of abnormal lung and heart parameters was lower, while in those with positive anti-Scl-70 antibodies the risk was increased. More-severe microangiopathy in the nailfold area correlated independently with cardiac and pulmonary involvement and in some cases revealed increased risk of cardiopulmonary manifestations, while the autoantibody status suggested low risk [19]. Tieu et al. (2018) performed semi-quantitative capillaroscopy (without quantitative measurement of capillary diameters and mean capillary density) and assessed the associations with the immunological profile in 152 SSc patients. Based on semiquantitave analysis, a total capillary damage score was calculated. Capillary dropout was assessed in four grades: 0 = normal; 1 + minimal/mild (involving less than 10% of the capillaries); 2 + moderate (10–50%); 3 + extensive/severe (greater than 50%). Patients positive for anti-RNAP III antibodies had a significantly higher nailfold-capillary total-damage-index as compared to those positive for CENP, Scl 70, or RNP. Patients with anti-Scl-70 or anti-RNAP III antibodies had greater dropout than cases with positive anti-centromere antibodies (although the disease duration was significantly shorter) [20].

As a novel observation in the current study, we found the presence of anti-RNAPIII–155 antibodies in patients with or without early microangiopathy. Our results differ from those of Tieu et al. (2018), who found greater capillary dropout in SSc patients positive for anti-RNAP III antibodies [20]. These differences could be associated with presence of different disease phenotypes in scleroderma patients positive for anti-RNAP antibodies. Considering complex disease pathogenesis and the existence of diverse clinical forms of SSc, it could be concluded that precise determination of the progression of microangiopathy and disease prognosis depends on multiple criteria. Interestingly, in SSc patients positive for anti-RNAP III (Callejas-Moraga et al., Spain, 26/221), it has been observed more frequently presence of symptoms different from RP at disease presentation. has been observed that the presence of symptoms is more often than not different from RP at disease presentation. In addition, diffuse skin involvement, scleroderma renal crisis, arthritis, and joint contractures were more frequent in patients positive for anti-RNAP antibodies [23,24]. Future observations from longitudinal studies could support the establishment of disease activity indices, which will combine data on immunological findings, capillaroscopic changes, severity of peripheral vascular syndrome, and other clinical criteria to better define different clinical forms of the disease—this would also facilitate the prediction of vascular and overall disease progression.

Of note, there are data that treatment with immunosuppressive drugs and bosentan could improve capillaroscopic changes in SSc patients. In the current study, only one of the patients with positive anti-RNAP III antibodies has received therapy with cyclophosphamide and corticosteroids in the last 6 months, and the absence in microangiopathy in this case might be related to effective treatment. None of the patients received bosentan. Two patients were on vasodilator therapy only, and one case with early microangiopathy received d-penicillamin, a vasodilator, and an anti-platelet drug [28,29,30].

Anti–RNAP III is one of the most frequent antinuclear antibodies in SSc after anti-centromere and anti–Scl-70 antibodies. The prevalence of anti-Scl-70 antibodies in SSc is estimated to be 20–35%, for anti-centromere antibodies 26–33%, and for anti–RNAP III antibodies 6–25%. Anti–RNAP III is not found in the same sera and is often mutually exclusive to anti–Scl-70 and anti-centromere antibodies [31,32,33,34].

Wielosz et al. (2020) have evaluated the prevalence of anti-RNAP III in 126 Polish SSc patients using the EUROLINE SSc profile. They observed 15% (19/126) frequency, epitope 11 present in 16 patients, and epitope 155 in 14 cases. In our group’s anti-RNAP III-antibody positive patients, 13 (68.4%) had no other SSc-associated antibodies. The diffuse cutaneous subtype, decreased diffusing capacity (DL_CO_), scleroderma renal crisis, and malignancy were more common than in anti-RNAP III-negative patients [35].

### 4.2. Associations between Capillaroscopic Changes and ANA Titer; Significance of AC Pattern

AC-5 or nuclear large/coarse speckled pattern (previously denoted as spliceosome/nuclear matrix pattern) is present to a varying degree in different systemic autoimmune rheumatic diseases (e.g., SSc, systemic lupus erythematosus, and mixed connective tissue disease) [36]. It is comprised of coarse speckles across the entire nucleoplasm. The nucleoli may be stained or not, and the chromatin mass of mitotic cells (metaphase, anaphase, and telophase) remains unstained (e.g., anti-Sm or anti-U1 RNP). In case of clinical suspicion for SSc, it is strongly advised to perform a follow-up test for anti-RNAP III antibodies (e.g., SSc profile) given that the anti-RNAP III antibodies are included in the classification criteria for SSc [2,3]. However, it is also essential to rule out the presence of other autoantibodies that demonstrate the AC-5 pattern on IIF (e.g., anti-hnRNPs, anti-U1RNP, and anti-Sm antibodies).

AC-10 or punctate nucleolar (previously known as a speckled nucleolar pattern) has been associated with anti-RNAP I autoantibodies [37,38,39]. While AC-10 is associated with anti-RNAP I antibodies, these antibodies almost always coexist with anti-RNAP III antibodies, which reveal the AC-5 pattern. Therefore, in case of clinical suspicion of SSc, it is highly recommended to perform further testing for anti-RNAP III antibodies, as specific immunoassays for anti-RNAP I antibodies are currently not commercially available.

## 5. Conclusions

In the current pilot study, the association between more-advanced capillaroscopic changes and the presence of anti-Scl-70 autoantibodies was confirmed. Thus, anti-Scl-70 autoantibodies may be related to the progression of microvascular pathology. As a novel observation, positive anti-RNAP III–155 antibodies were found in SSc patients with or without early microangiopathy. Based on the available data, it can be concluded that disease-specific autoantibodies are related to the degree of microangiopathy in SSc (although they are not the only factor that contributes to the progression of microvascular pathology). Different phenotypes of clinical involvement and vascular damage may exist in patients with the same disease-specific autoantibody. Studying the association between capillaroscopic changes with diagnostic and pathogenic autoantibodies in SSc is expected to improve current knowledge about pathogenesis, classification, and prognosis of the disease.

## Figures and Tables

**Table 1:** Demographic and clinical characteristics of the patients.

	SSc Patients, *n* = 19
Gender	1 male, 18 females
Age	51.56 ± 15.07 years
Type of cutaneous involvement	16 limited, 3 diffuse cutaneous involvement
Presence of RP Digital ulcers	100% 57% (11/19)
Other manifestations:	
Pulmonary Cardiac Joint Tendon	57% (11/19) 10.5% (2/19) 42% (8/19) 21% (4/19)
Treatment in the last 6 months:	
Vasodilators Antiplatelet drugs Low-dose corticosteroids Cytotoxic drugs D-penicillamine	79% (15/19) 26% (5/19 26% (5/19) 15.7% (3/19) 15.7% (3/19)

**Table 2:** Characteristics of patients with positive anti-RNAP III–155 antibody.

Case 1	20-year old male patient with limited cutaneous involvement, RP, joint and tendon involvement. Disease duration: 5 years. ANA titer 1:320, AC-5. The patient has received vasodilators in the last 6 months. Capillaroscopic examination revealed normal pattern.
Case 2	64-year old female patient with diffuse cutaneous involvement, RP, pulmonary involvement. Disease duration: 14 years. ANA titer 1:320, AC-5. The patient has received therapy with cyclophosphamide and corticosteroids in the last 6 months. The patient has received vasodilators, anti-platelet drug, and D-penicillamin in the last 6 months. Capillaroscopic examination revealed normal pattern.
Case 3	57-year old female patient with limited cutaneous involvement, RP, joint and tendon involvement. Disease duration: 13 years. ANA titer 1:160, AC-5. Capillaroscopic examination revealed “early” pattern.
Case 4	32-year old female patient with limited cutaneous involvement, RP, joint and tendon involvement. Disease duration: 15 years. ANA titer 1:1280, AC-5, AC-10. The patient has received vasodilators in the last 6 months. Capillaroscopic examination revealed normal pattern.

## Data Availability

Not applicable.
